# Supplementary material for: Nasopharyngeal carriage, antimicrobial susceptibility patterns, and associated factors of Gram-positive bacteria among children attending the outpatient department at the University of Gondar Comprehensive Specialized Hospital, Northwest Ethiopia
Source: PLoS One. 2024 Aug 28;19(8):e0308017. doi: 10.1371/journal.pone.0308017 (PMC11357108; doi:10.1371/journal.pone.0308017)
Supplement: Attachment — Submitted filename: Reviwers commnets to PLOS ONE.docx [file pone.0308017.s001.docx]

**Manuscript Number: PONE-D-24-10619**

**Manuscript Title: Nasopharyngeal Carriage, Antibiotic Susceptibility Patterns, and Associated Factors of Gram-positive Bacteria among children attending the outpatient department at the University of Gondar Comprehensive Specialized Hospital, Northwest Ethiopia**

**Dear editor,**

**I would like to say thanks for giving the opportunity and the invitation. It was my pleasure to review this scientific paper. I have provided comments, reconditions, and question to the authors below:**

**Point by Point comments to Authors**

1. The main concern of this paper is it lacks novelty. There are many researches in Ethiopia in general and even at University of Gondar Comprehensive Specialized Hospital in particular on the Nasopharyngeal Carriage and Antibiotic Susceptibility Patterns of Gram-positive isolates: (DOI:[10.1016/j.pedneo.2013.03.017](http://dx.doi.org/10.1016/j.pedneo.2013.03.017)DOI: [10.1016/j.pedneo.2013.03.017](https://doi.org/10.1016/j.pedneo.2013.03.017),  [**https://doi.org/10.3390/children4040027**](https://doi.org/10.3390/children4040027), <https://doi.org/10.1177/23333928231186687>, DOI:[10.4172/2332-0877.1000109](http://dx.doi.org/10.4172/2332-0877.1000109), DOI: [10.2147/IDR.S385866](https://doi.org/10.2147/idr.s385866)…..). Thus, it is better to supplement some important issues like genotypic characterizations of the isolates via extended your study time, and the study groups (please include the inpatients).
2. Why you focus only on Gram-positive bacteria? For example Gram negative bacteria like Haemophilus influenzae, Klebsiella pneumoniae, Micrococcus catarrhalis ………. Can induce Nasopharyngeal Carriage
3. What about the viral and the fungal agents? As there are some fungal and viral agents, which cause Nasopharyngeal Carriage (DOI: [10.1186/s41479-021-00088-5](https://doi.org/10.1186/s41479-021-00088-5), <https://pubmed.ncbi.nlm.nih.gov/23798081/>)
4. Line number 32. Please convert Epi-Data version 4.6.0.6 to 4.6
5. Line number 43: Please convert risk factors to associated factors
6. In the abstract section, minimize the conclusions and the recommendations and omit recommendation as sub-title in the abstract part.
7. In line number 50, please remove colon (:) in front of the introduction
8. The introduction section needs some modification as it focuses on the scientific background. Hence, it will be grateful if you start from the problem with respect to the WHO and CDC data.
9. Please state the problem and prevalence of nasopharyngeal carriage and drug resistance profiles of the isolates among children in Ethiopia and the study area in figures?
10. Online number 105, please convert general objectives to general objective?
11. Why you excluded under 15 ages Children and those with a history of nasal surgery, respiratory infection, and antibiotics from the study?
12. You have used a single population proportion formula to calculate the sample size. Thus, please write the formula and how to obtained 424 and why you used 50% proportion? I think there are plenty of articles even in your study area.
13. Who analyzed the data? You said a trained laboratory technologist analyzed the data (on line numbers 155 and 156).
14. On line number 238, please change p-value ≤ 0.2 to 0.25
15. Please omit the natural resistance profiles of the isolates (those which have known resistance profile to certain antibiotics). For instance, S. aureus naturally resistant to penicillin, clindamycin, and azithromycin.
16. Online number 305, please covert 257 to text as it comes before the full stop.
17. In the discussion section, please state the scientific justifications to associate factors
18. It is better if you minimize the Conclusions and Recommendations statements as it is so bulky
19. Online 461 and 462, avoid the figure (69.8%) and write as low, intermediate, and high
20. In the authors’ contributions statement, please avoid the bolded letters
21. Please re-write the competing of interest and funding statement
22. There are many too old references in the manuscript (ref 2, 3, 4, 18, 20, 25, 27, 30, 31, 34, 41, 44, 49, 54, 68, 69, 76, 78…..). Hence, please update and replace them.

**Question**

1. What is the clinical utility and value of your study? In your study, the most frequently identified isolate was S. aureus. However, S. aureus is the known causative agent for Nasopharyngeal Carriage. Moreover, Gram-positive bacteria, including S. aureus, Streptococcus pyogens, and Streptococcus pneumoniae are the cause of Nasopharyngeal Carriage naturally. Thus, what is the importance doing this research?
