# Supplementary material for: Nasopharyngeal carriage, antimicrobial susceptibility patterns, and associated factors of Gram-positive bacteria among children attending the outpatient department at the University of Gondar Comprehensive Specialized Hospital, Northwest Ethiopia
Source: PLoS One. 2024 Aug 28;19(8):e0308017. doi: 10.1371/journal.pone.0308017 (PMC11357108; doi:10.1371/journal.pone.0308017)
Supplement: Attachment — Submitted filename: Response to Reviewers.docx [file pone.0308017.s002.docx]

**Rebuttal Letter**

**Date**: June 15, 2024

**Original Manuscript ID:** PONE-D-24-10619

**Original Article Title:** Nasopharyngeal Carriage, Antibiotic Susceptibility Patterns, and Associated Factors of Gram-positive Bacteria among children attending the outpatient department at the University of Gondar Comprehensive Specialized Hospital, Northwest Ethiopia

**To**: PLOS ONE

**Re**: Response to academic editor and reviewers

Dear Editor,

Thank you for allowing a resubmission of our manuscript, with an opportunity to address the academic editor and reviewers’ comments.

We are uploading

(1) Our point-to-point response to the comments below (response to academic editor and reviewer(s), respectively)

(2) A marked-up copy with track changes (Revised Manuscript with Track Changes)

(3) An unmarked version without track changes (Manuscript)

Best regards,

<Abebe Birhanu> et al

**NOTE: We have further addressed the Journal Requirements as follows:**

**Concern #1.** Please ensure that your manuscript meets PLOS ONE's style requirements, including those for file naming. The PLOS ONE style templates can be found at 
<https://journals.plos.org/plosone/s/file?id=wjVg/PLOSOne_formatting_sample_main_body.pdf> and 
<https://journals.plos.org/plosone/s/file?id=ba62/PLOSOne_formatting_sample_title_authors_affiliations.pdf>

**Authors’ response**: We have adhered to PLOS ONE's style and format requirements in the revised manuscript.

**Concern #2.** We found significant text overlap between your submission and previous work in the (introduction, conclusion). Please revise the manuscript to rephrase the duplicated text, cite your sources, and provide details as to how the current manuscript advances on previous work.

**Authors’ response**: We have revised the manuscript to address these concerns, rephrasing the duplicated text, citing sources appropriately, and detailing the advancements made by the current work.

**Concern #3.** Upon submission, please confirm that the manuscript, or any related manuscript, is not currently under consideration or accepted elsewhere. If related work has been submitted to PLOS ONE or elsewhere, authors must include a copy with the submitted article. Reviewers will be asked to comment on the overlap between related submissions (<http://journals.plos.org/plosone/s/submission-guidelines#loc-related-manuscripts)>.

**Authors’ response**: We confirm that the manuscript, or any related manuscript, is not under consideration or accepted elsewhere.

**Concern #4.** Thank you for stating the following in your Competing Interests section:  
"The authors' have no conflict of interest." Please complete your Competing Interests on the online submission form to state any Competing Interests. If you have no competing interests, please state "The authors have declared that no competing interests exist.", as detailed online in our guide for authors at <http://journals.plos.org/plosone/s/submit-now>. This information should be included in your cover letter; we will change the online submission form on your behalf.

**Authors’ response**: We have included the statement "The authors have declared that no competing interests exist" in the revised manuscript and cover letter.

**Authors' Response to Additional Editor Comments**

Dear Editor, we are grateful for the opportunity to revise our manuscript for PLOS ONE and for the insightful comments from the reviewers, which have significantly improved our work.

**Editor Comment**: Although what authors are reporting is not a novelty, they must however be commended for their efforts in reporting what is prevailing in their locality.

**Authors' Response**: Thank you for your positive feedback.

**Editor Comment**: The email addresses of authors were not needed…. except the corresponding author.

**Authors' Response**: We have updated the manuscript accordingly.

**Editor Comment**: Based on PLOS ONE authors’ guidelines, figures must be uploaded separately in TIF or another form.

**Authors' Response**: As requested, we have uploaded the figures in the specified format.

**Editor Comment**: Line 27: …It is better written as, ‘…was conducted from May 1, 2023 to August 30, 2023.’

**Authors' Response**: We have revised the text to "was conducted from May 1, 2023, to August 30, 2023."Please refer to line 28 in the revised manuscript.

**Editor Comment**: Lines 29 & 30: ‘Bacterial species were identified by biochemical tests.’ It is rewritten better as, ‘Bacterial species were identified by colony morphology, Gram stain and biochemical tests.’

**Authors' Response**: We have revised the text to: "Bacterial species were identified by colony morphology, Gram stain, and biochemical tests." Please refer to lines 30-31 in the revised manuscript.

**Editor Comment**: Lines 31 & 32: ‘…… were determined using the cefoxitin disc and D-test, respectively.’ Do you think ‘cefoxitin disc and D-test’ are equivalent terms? Drug vs. technique?

**Authors' Response**: We have corrected this to: "D-tests were conducted using clindamycin and erythromycin discs to detect inducible clindamycin resistance, while cefoxitin disc tests were utilized to ascertain methicillin resistance." Please refer to lines 32-34 in the revised manuscript.

**Editor Comment**: Lines 46: Write their long form of the ‘MRSA and MDR’

**Authors' Response**: We have corrected the text to "methicillin-resistant *S. aureus* and multi-drug resistant." Please refer to line 50 in the revised manuscript.

**Editor Comment**: The abstract should be rewritten and make adjustments based on the comments in the methods and results part that follows.

**Authors' Response**: We have revised the abstract accordingly.

**Editor Comment**: Once you have used the long form of the name of the bacterium then you can abbreviate the genus name throughout the body of the paper, except headings and subheadings.

**Authors' Response**: We have made the necessary corrections throughout the manuscript.

**Editor Comment**: Lines 104 to 115…. remove the objective.

**Authors' Response**: We have removed the objective as requested.

**Editor Comment**: Line 123: revise the total population of the town. It is expected above one million.

**Authors' Response**: We have revised the population estimate to 2,929,628. Please refer to line 122 in the revised manuscript.

**Editor Comment**: A separate subheading is needed as, ‘Study population’

**Authors' Response**: We have added a separate subheading for "Study population." Please refer to line 125 in the revised manuscript.

**Editor Comment**: A separate subheading for ‘Sample size determination and sampling technique’

**Authors' Response**: We have added a separate subheading for "Sample size determination and sampling technique." Please refer to line 131 in the revised manuscript.

**Editor Comment**: Remove variables of the study (lines 140-152).

**Authors' Response**: We have deleted the variables of the study.

**Editor Comment**: Why did you fail to include chocolate agar, especially for isolating fastidious organisms like *S. pneumoniae*?

**Authors’ Response**: We acknowledge that chocolate agar is a superior medium for the growth of all types of bacteria, including fastidious organisms. Blood agar, on the other hand, is also effective for cultivating both Gram-positive and Gram-negative bacteria, including some fastidious species such as *S. pneumoniae* and *S. pyogenes*. However, unlike chocolate agar, blood agar allows for the observation of hemolysis patterns, which is crucial for identifying whether *S. pneumoniae* is alpha, beta, or gamma hemolytic.

**Editor Comment**: What is the advantage of performing oxacillin and cefoxitin disc diffusion tests concurrently? Can an oxacillin susceptibility test be performed by disc diffusion for *S. aureus*?

**Authors’ Response**: Thank you for your insightful comment. Indeed, the CLSI 2022 does not recommend using the oxacillin disc diffusion method for determining the susceptibility of *S. aureus*. However, we used a 1µg oxacillin disc specifically to assess the non-susceptibility of *S. pneumoniae* to penicillin, not for *S. aureus*. In our study, 1µg oxacillin and 30µg cefoxitin discs were used to determine the non-susceptibility of *S. pneumoniae* to penicillin and to identify methicillin-resistant *S. aureus*, respectively. Please refer to lines 225-226 in the revised manuscript for more details.

**Editor Comment**: Why did you omit 'vancomycin susceptibility testing', the preferred drug for MRSA?

**Authors’ Response**: We appreciate this important comment. While vancomycin is indeed the last-line treatment for MRSA, the CLSI 2022 guidelines recommend using minimum inhibitory concentration (MIC) methods, such as the E-test, for vancomycin susceptibility testing in *S. aureus*. However, due to the unavailability of the E-test in our laboratory, we conducted antibiotic susceptibility testing using the disc diffusion method for all identified isolates in this study.

**Editor Comment**: Some results require both the numerator and denominator. Similarly, associated factor assessment data requires a P-value.

**Authors’ Response**: Thank you for your feedback. We have made the necessary corrections to include both the numerator and denominator where applicable, and we have added P-values for the assessment of associated factors.

**Editor Comment**: All table titles should be complete in description, area, and period of study.

**Authors’ Response**: We have reviewed and revised the table titles to ensure they are complete and include the description, area, and period of the study.

**Editor Comment**: Many of the reasons for variation discussed were already extensively covered by other authors.

**Authors’ Response**: We have addressed this by supplementing our discussion with additional information to explain the variations, ensuring our work contributes new insights.

**Editor Comment**: The conclusion was beyond your scope. Please revise it based on community/government importance, focusing on the most significant findings.

**Authors’ Response**: We appreciate this valuable feedback and have revised our conclusion to align more closely with our findings. We emphasize the importance of our results in raising community awareness about bacterial transmission and informing infection prevention and control strategies in hospitals.

**Editor Comment**: Lines 479-484: Remove authors' contributions; the system generates this automatically.

**Authors’ Response**: We have accepted this suggestion and removed the authors' contributions section as requested.

**Editor Comment**: The manuscript did not appropriately follow the PloS One writing protocol and the binomial nomenclature for bacterial names.

**Authors’ Response**: Thank you for highlighting this issue. We have revised the manuscript to adhere to the PloS One writing guidelines and properly use the binomial nomenclature for bacterial names.

**Authors’ Response to Reviewer #1:**

First, we would like to extend our gratitude to the reviewers for their critical, constructive, and timely comments. To facilitate your review, we have copied your comments below alongside our responses. We have addressed all questions and concerns point by point as follows:

**Reviewer #1 Comment**: The paper lacks novelty. There have been many studies in Ethiopia, including your study area, on Nasopharyngeal Carriage and Antibiotic Susceptibility Patterns of Gram-positive isolates. It would be beneficial to include additional important aspects such as genotypic characterizations of the isolates, extended study periods, and broader study groups, including inpatients.

**Author’s Response**: While the study may seem to lack novelty, it provides comprehensive information not covered in previous studies. For instance, previous research at the University of Gondar Comprehensive Specialized Hospital focused solely on *S. pneumoniae* and did not report on the inducible clindamycin resistance profiles of isolates. This study, however, highlights the overall carriage state of Gram-positive bacteria and includes their resistance profiles, including inducible clindamycin resistance. Although we stored samples at -80°C, we were unable to perform genotypic characterization due to reagent shortages. Furthermore, we excluded inpatient children for two reasons:

1. Many inpatient children are already receiving treatment, which could lead to false-negative results if we sampled them.

2. Sampling from untreated inpatient children could yield artificially high carriage rates due to the hospital environment, resulting in false-positive results.

**Reviewer #1 Comment**: Why focus only on Gram-positive bacteria? Gram-negative bacteria, such as *Haemophilus influenzae, Klebsiella pneumoniae,* and *Moraxella catarrhalis*, can also induce nasopharyngeal carriage.

**Authors’ Response**: The comment is valid, and we recognize the role of some Gram-negative bacteria in the nasopharyngeal carriage. However, due to resource limitations and the broad scope of the title, we focused our study on Gram-positive bacteria.

**Reviewer #1 Comment**: What about viral and fungal agents? There are fungal and viral agents that cause nasopharyngeal carriage (DOI: 10.1186/s41479-021-00088-5, https://pubmed.ncbi.nlm.nih.gov/23798081/).

**Authors Response**: While this is a significant comment, identifying viruses and fungi in our laboratory setting in Ethiopia is challenging due to the lack of advanced techniques and necessary reagents.

**Reviewer #1 Comment**: Line number 32: Please convert Epi-Data version 4.6.0.6 to 4.6.

**Authors’ Response**: Yes, we have accepted and corrected this. Please refer to line 35 in the revised manuscript.

**Reviewer #1 Comment**: Line number 43: Please convert "risk factors" to "associated factors".

**Authors’ Response**: Yes, we have accepted and corrected this. Please refer to line 44 in the revised manuscript.

**Reviewer #1 Comment**: In the abstract section, minimize the conclusions and recommendations and omit recommendations as a sub-title. Please refer to lines 49-52 in the revised manuscript.

**Authors’ Response**: Yes, we have accepted and corrected this.

**Reviewer #1 Comment**: In line number 50, please remove the colon (:) in front of the introduction.

**Authors’ Response**: Yes, we have accepted and corrected this. Please refer to line 54 in the revised manuscript.

**Reviewer #1 Comment**: The introduction section needs some modification as it focuses too much on the scientific background. It would be better to start with the problem.

**Authors’ Response**: We have revised the introduction to include additional information. Please refer to line 93 in the revised manuscript.

**Reviewer #1 Comment**: Please state the problem and prevalence of nasopharyngeal carriage and drug resistance profiles of the isolates among children in Ethiopia and the study area using figures.

**Authors’ Response**: Yes, we have accepted and corrected this. Please refer to lines 94-97 in the revised manuscript.

**Reviewer #1 Comment**: On line number 105, please convert "general objectives" to "general objective".

**Authors’ Response**: Yes, we have accepted and deleted this because it was also raised by the academic editor.

**Reviewer #1 Comment**: Why exclude children under 15 and those with a history of nasal surgery, respiratory infection, or antibiotic use from the study?

**Authors’ Response**: Exclusion criteria were generated to ensure accurate results. Including children with a history of nasal surgery, signs of respiratory infection, and recent antibiotic use could lead to nose obstruction, overestimated results, and underestimated results, respectively.

**Reviewer #1 Comment**: Please write the formula and explain how you obtained 424 and why you used a 50% proportion. There are many relevant articles, even in your study area.

**Authors’ Response**: We have accepted this comment and included the formula in the revised manuscript. Please refer to lines 140-150 in the revised manuscript. We used a 50% proportion for the following reasons:

1. Using the prevalence from Assefa et al., 2013, would be outdated and may not reflect the current population, leading to a low sample size.

2. The prevalence from Belayhun et al., 2023, was not used due to population and geographical differences, which would also result in a low sample size.

3. Using a 50% prevalence ensures a larger sample size, which is beneficial for statistical analysis and result interpretation.

**Reviewer #1 Comment**: Who analyzed the data? You mentioned that a trained laboratory technologist analyzed the data (on line numbers 155 and 156).

**Authors’ Response**: Thank you for your suggestion. A trained laboratory technologist collected the data, and the analysis was carried out with the assistance of a senior biostatistician. Please refer to lines 153-155 in the revised manuscript.

**Reviewer #1 Comment**: On line number 238, please change the p-value ≤ 0.2 to 0.25.

**Authors’ Response**: Yes, we have revised and corrected it accordingly. Please refer to line 242 in the revised manuscript.

**Reviewer #1 Comment**: Please omit the natural resistance profiles of the isolates (those with known resistance profiles to certain antibiotics). For example, *S. aureus* is naturally resistant to penicillin, clindamycin, and azithromycin.

**Authors’ Response**: Although resistance to these antibiotics has increased, they were historically used as treatment options for *S. aureus* infections. For instance, penicillin was extensively and effectively employed for treating *S. aureus* infections in the 1940s and 1950s [1]. In addition, clindamycin is currently a reserve drug of choice for treating methicillin-resistant *S. aureus* (MRSA) infections [2]. Thus, the resistance observed is due to excessive use of these antibiotics, horizontal gene transfer, and enzymatic production [3]. Moreover, the Clinical and Laboratory Standards Institute (CLSI) 2022 guidelines recommend performing disk diffusion for antibiotic susceptibility testing of *S. aureus* against penicillin, clindamycin, and azithromycin [4].

**Reviewer #1 Comment**: On line number 305, please convert 257 to text as it comes before the full stop.

**Authors’ Response**: Yes, we have rewritten and corrected it. Please refer to lines 312-313 in the revised manuscript.

**Reviewer #1 Comment**: In the discussion section, please state the scientific justifications for associating factors.

**Authors’ Response**: We appreciate this suggestion and have supplemented additional scientific justifications in the revised manuscript. Please refer to lines 457 and 463-465 in the revised manuscript.

**Reviewer #1 Comment**: It is better if you minimize the conclusions and recommendations as they are too lengthy.

**Authors’ Response**: Yes, we have accepted and corrected this. Please refer to lines 474-487 in the revised manuscript.

**Reviewer #1 Comment**: On lines 461 and 462, avoid the figure (69.8%) and write as low, intermediate, and high.

**Authors’ Response**: Yes, we have accepted and corrected this. Please refer to line 474 in the revised manuscript.

**Reviewer #1 Comment**: In the authors' contributions statement, please avoid the bolded letters.

**Authors’ Response**: Yes, we have accepted and deleted this because it was also commented on by the academic editor.

**Reviewer #1 Comment**: Please rewrite the competing interests and funding statement.

**Authors’ Response**: Yes, we have accepted and corrected this. Please refer to lines 495 and 497 in the revised manuscript.

**Reviewer #1 Comment**: Please update and replace outdated references in the manuscript (e.g., ref 2, 3, 4, 18, 20, 25, 27, 30, 31, 34, 41, 44, 49, 54, 68, 69, 76, 78, etc.).

**Authors’ Response**: We appreciate your feedback and have updated the references accordingly.

**Reviewer #1 Comment**: What is the clinical utility and value of your study? In your study, the most frequently identified isolate was *S. aureus*, which is a known causative agent for nasopharyngeal carriage. Gram-positive bacteria, including *S. aureus, S. pyogenes*, and *S. pneumoniae*, naturally cause nasopharyngeal carriage. Thus, what is the importance of conducting this research?

**Authors’ Response**: Nasopharyngeal carriage of these bacteria is a common occurrence in young children. However, the worst thing is that nasopharyngeal carriage serves as a key factor in the development of severe respiratory and invasive bacterial infections like otitis media, sinusitis, pneumonia, bacteremia, and meningitis. These infections occur because the nasopharynx is borderline with bacteria-limited sites like the lower respiratory tract, middle ear, and sinuses, making it a source of autoinfection and transmission to others, despite being initially asymptomatic [5-8]. These infections are major contributors to pediatric and neonatal deaths globally [9], the most frequent cause of under-5 children seeking medical care in the United States [10], and the second-most prevalent reason for the overprescription of antibiotics [11]. Additionally, monitoring and updating the antibiotic susceptibility patterns of these isolates is essential as drug resistance becomes an increasing public health concern.

**Authors’ response to Reviewer #2:**

**Reviewer #2 General Comment**: The study mixes carriage isolates with what are likely to be clinical isolates.

**Authors’ Response**: Thank you for your insightful comment. We have made the necessary corrections based on your suggestion. Please refer to lines 412-415 in the revised manuscript.

**Reviewer #2 General Comment**: Why do you exclude Gram-negative bacteria?

**Authors’ Response**: Including Gram-negative bacteria would indeed strengthen our study. However, due to material constraints and feasibility issues, we were unable to isolate Gram-negative bacteria.

**Reviewer #2 General Comment**: Uniform writing is needed for the names of drugs.

**Authors’ Response**: We appreciate your attention to this detail. We have made the required corrections to ensure uniformity in the revised manuscript.

**Reviewer #2 General Comment**: The use of the English language is poor in certain sections and would require a detailed revision.

**Authors’ Response**: We have improved the use of the English language throughout the manuscript with the help of local editors.

**Abstract**

**Reviewer #2 Comment**: Lines 32-34: 'The data was entered into Epi-Data version 4.6.0.6 and exported to SPSS version 25 for analysis. The adjusted odds ratio at a 95% confidence interval with a P-value of < 0.05 was taken as statistically significant.' It is better written as 'The data was entered into Epi-Data version 4.6.0.6 and exported to SPSS version 25 for analysis. Bivariable and multivariable logistic regression analysis was used to identify associated risk factors. The adjusted odds ratio at a 95% confidence interval with a P-value of < 0.05 was taken as statistically significant.'

**Authors’ Response**: We have revised the text to include "Bivariable and multivariable logistic regression analyses were employed to identify associated factors." Please refer to lines 36-37 in the revised manuscript.

**Reviewer #2 Comment**: The number of isolates with the total is better written together with the percentages. Resistant/susceptible with the total including the percentage also should be used. This works for the whole abstract and results part of the paper. For example, Lines 35 & 36: 'The overall nasopharyngeal carriage of Gram-positive bacteria was 296 (69.8%, 95% CI: 65.3–74.0). Of these, *S. aureus* constitutes 122 (28.8%), followed by *S. pneumoniae* 92 (21.7%).' It is better written as, 'The overall nasopharyngeal carriage of Gram-positive bacteria was 296/424 (69.8%, 95% CI: 65.3–74.0). Of these, *S. aureus* constitutes 122/424 (28.8%), followed by *S. pneumoniae* 92/424 (21.7%).'

**Authors’ Response**: We have revised the abstract and results section accordingly to include both the numbers and percentages. Please refer to lines 39-44 in the revised manuscript.

**Reviewer #2 Comment**: Lines 40-43: P-value is needed.

**Authors’ Response**: We have included the P-value as suggested. Please refer to lines 45-48 in the revised manuscript.

**Reviewer #2 Comment**: Line 43: …… risk factors……. It is better written as……. associated factors…….

**Authors’ Response**: We have replaced 'risk factors' with 'associated factors.' Please refer to line 44 in the revised manuscript.

**Introduction**

**Reviewer #2 Comment**: Once you have used the long form of the name of the bacterium then you can abbreviate the genus name throughout the body of the paper, except headings and subheadings.

**Authors’ Response**: We appreciate this suggestion and have made the appropriate corrections throughout the revised manuscript.

**Materials and Methods**

**Reviewer #2 Comment**: Line 53: Remove the word ‘analysis’

**Authors’ Response**: We have removed the word 'analysis.

**Reviewer #2 Comment**: Specimen collection and processing needs to be well written.

**Authors’ Response**: We have revised the section on specimen collection and processing to provide a clearer and more detailed description. Please refer to lines 157-165 in the revised manuscript.

**Reviewer #2 Comment**: What standard microbiological procedures were employed in specimen collection, isolation, identification, susceptibility testing, and throughout the procedure?

**Authors’ Response**: This is a good comment for us. The revised manuscript is supported with “The standard microbiological procedures for species identification of bacteria in our work were Gram-stain, morphological appearances (culture), and biochemical tests. Kirby-Bauer disk diffusion and modified Kirby-Bauer disk diffusion methods on the Mueller-Hinton agar plate were standard microbiological procedures for antibiotic susceptibility testing”. Please refer to lines 175-176 and 208-212 in the revised manuscript.

**Reviewer #2 Comment**: How could you standardize the suspension preparation while performing antimicrobial susceptibility testing?

**Authors’ Response**: Thank you for your constructive feedback. We have added the following to the revised manuscript: "To standardize the bacterial suspension preparation, 0.85% normal saline was used. If the suspension was more turbid than the 0.5 McFarland standard, it was diluted accordingly. Conversely, if the suspension was less turbid, additional pure colonies of the target bacteria were added to achieve the correct turbidity." Please refer to lines 203-206 in the revised manuscript.

**Reviewer #2 Comment**: What is the advantage of using cefoxitin disc diffusion over oxacillin disc for susceptibility testing for methicillin-resistant *S. aureus* (MRSA)?

**Authors’ Response**: We appreciate your comment. Cefoxitin is preferred in disk diffusion testing because it provides clearer results and is a more sensitive and specific indicator of mecA-mediated methicillin resistance compared to oxacillin [12, 13].

**Reviewer #2 Comment**: It is better to use 'Data analysis' rather than 'Statistical analysis.'

**Authors’ Response**: Yes, we have replaced 'Statistical analysis' with 'Data analysis.' Please refer to line 151 in the revised manuscript.

**Reviewer #2 Comment**: What measures were taken for children who tested positive for the microorganisms?

**Authors’ Response**: Thank you for pointing this out. Children who carried Gram-positive bacteria were reported to physicians for appropriate medical care. Please refer to lines 253-254 in the revised manuscript.

**Results**

**Reviewer #2 Comment**: How can you define community-associated methicillin-resistant *S. aureus* (CA-MRSA)? How can you exclude nosocomial sources?

**Authors’ Response**: Thank you for this valuable comment. CA-MRSA infections are defined as MRSA infections in individuals who have not had any of the following risk factors within the year before the MRSA culture date [14]:

1. Surgery or hospitalization

2. Permanent indwelling catheters or percutaneous medical devices

3. Living in an inpatient care institution

4. Dialysis

5. MRSA culture within 48 hours after hospital admission.

While molecular methods are the confirmatory tests to differentiate between nosocomial and community-acquired MRSA, these are not available in our laboratory. However, nosocomial infections are generally defined as infections occurring 48 hours or more after hospital admission or within 30 days of discharge from the inpatient department [15]. In our study, however, the participants were outpatient children who visited the hospital and returned home within 24 hours.

**Reviewer #2 comment**: What is your base for classifying the age group? Have you associated with possible risks of interest? It is better again to re-group and perform the analysis.

**Authors’ response**: We thank you for bringing this issue to our attention. After reviewing different articles related to our work, we have tried to regroup the ages of the study participants based on their level of exposure to the nasopharyngeal carriage of Gram-positive bacteria. Please refer to lines 264 (Table 1) and 341(Table 5) in the revised manuscript.

**Discussions**

**Reviewer #2 comment**: The authors tried to compare their findings with different reports from worldwide. They also better justify the reason for variation among results of different research findings concerning theirs’ based on actual situations.

**Authors’ response**: We thank you for giving good feedback.

**Conclusions**

**Reviewer #2 comment**: Normally, the conclusion emanates from the research.

**Authors’ response**: We appreciate your positive feedback.

Thank you very much for your valuable time to review and comment on our manuscript.

Authors

**References:**

1.	Rungelrath V, DeLeo FR. *Staphylococcus aureus*, Antibiotic Resistance, and the Interaction with Human Neutrophils. *Antioxid Redox Signal*. 2021;**34**(6):452-70.

2.	Ambachew A, Gebrecherkos T, Ayalew G. Prevalence and Clindamycin Resistance Profile of *Staphylococcus aureus* and Associated Factors among Patients Attending the University of Gondar Comprehensive Specialized Hospital, Gondar, Northwest Ethiopia. *Interdiscip Perspect Infect Dis*. 2022;**2022**:6503929.

3.	Vestergaard M, Frees D, Ingmer H. Antibiotic Resistance and the MRSA Problem. *Microbiol Spectr*. 2019;7(2).

4.	CLSI. Performance Standards for Antimicrobial Susceptibility Testing M100. 32nd edition 2022.

5.	Flynn M, Dooley J. The microbiome of the nasopharynx. *J Med Microbiol*. 2021;**70**(6):001368.

6.	Kovács E, Sahin-Tóth J, Tóthpál A, van der Linden M, Tirczka T, Dobay O. Co-carriage of *Staphylococcus aureus, Streptococcus pneumoniae, Haemophilus influenzae* and *Moraxella catarrhalis* among three different age categories of children in Hungary. *PloS One*. 2020;**15**(2):e0229021.

7.	Navne JE, Børresen ML, Slotved HC, Andersson M, Melbye M, Ladefoged K, et al. Nasopharyngeal bacterial carriage in young children in Greenland: a population at high risk of respiratory infections. *Epidemiol Infect*. 2016;**144**(15):3226-36.

8.	Gámez G, Rojas JP, Cardona S, Castillo JD, Palacio MA, Mejía LF, et al. Nasopharyngeal Colonization, Associated Factors and Antimicrobial Resistance of *Streptococcus pneumoniae* among Children under 5 Years of Age in the Southwestern Colombia. 2020:2020.04. 09.20058529.

9.	Qazi S, Aboubaker S, MacLean R, Fontaine O, Mantel C, Goodman T, et al. Ending preventable child deaths from pneumonia and diarrhoea by 2025. Development of the integrated Global Action Plan for the Prevention and Control of Pneumonia and Diarrhoea. *Arch Dis Child*. 2015;**100** Suppl 1:S23-8.

10.	Donnelly JP, Baddley JW, Wang HE. Antibiotic utilization for acute respiratory tract infections in U.S. emergency departments. *Antimicrob Agents Chemother*. 2014;**58**(3):1451-7.

11.	Dekker AR, Verheij TJ, van der Velden AW. Inappropriate antibiotic prescription for respiratory tract indications: most prominent in adult patients. *Fam Pract*. 2015;**32**(4):401-7.

12.	Bonjean M, Hodille E, Dumitrescu O, Dupieux C, Nkoud Mongo C, Allam C, et al. Disk Diffusion Testing for Detection of Methicillin-Resistant Staphylococci: Does Moxalactam Improve upon Cefoxitin? *J Clin Microbiol*. 2016;**54**(12):2905-9.

13.	Centers for Disease Control and Prevention. Laboratory Testing for Methicillin (oxacillin)-resistant Staphylococcus aureus (MRSA) 2024 [cited May 21, 2024 May 21, 2024]. Available from: https://[www.cdc.gov/mrsa/php/laboratories/index.html#:~:text=Cefoxitin%20is%20a%20better%20inducer,the%20historic%20role%20of%20methicillin](http://www.cdc.gov/mrsa/php/laboratories/index.html#:~:text=Cefoxitin%20is%20a%20better%20inducer,the%20historic%20role%20of%20methicillin).

14.	Community-associated Methicillin-resistant Staphylococcus aureus (CA-MRSA):

Fact Sheet for Healthcare Providers 2022 [Available from: https://www.health.state.mn.us/diseases/staph/mrsa/mrsahealthcare.html#:~:text=The%20majority%20of%20CA%2DMRSA,infections%2C%20necrotizing%20pneumonia%20or%20septicemia.

15.	Revelas A. Healthcare - associated infections: A public health problem. *Niger Med J*. 2012;**53**(2):59-64.
