# Supplementary material for: Nasopharyngeal carriage, antimicrobial susceptibility patterns, and associated factors of Gram-positive bacteria among children attending the outpatient department at the University of Gondar Comprehensive Specialized Hospital, Northwest Ethiopia
Source: PLoS One. 2024 Aug 28;19(8):e0308017. doi: 10.1371/journal.pone.0308017 (PMC11357108; doi:10.1371/journal.pone.0308017)
Supplement: Attachment — Submitted filename: Rco to authors. Plose One.docx [file pone.0308017.s003.docx]

As much as possible, the authors address and cover my concern.
