# Supplementary material for: Nasopharyngeal carriage, antimicrobial susceptibility patterns, and associated factors of Gram-positive bacteria among children attending the outpatient department at the University of Gondar Comprehensive Specialized Hospital, Northwest Ethiopia
Source: PLoS One. 2024 Aug 28;19(8):e0308017. doi: 10.1371/journal.pone.0308017 (PMC11357108; doi:10.1371/journal.pone.0308017)
Supplement: Attachment — Submitted filename: review.docx [file pone.0308017.s004.docx]

**Title: -** Nasopharyngeal Carriage, Antibiotic Susceptibility Patterns, and Associated Factors of Gram-positive Bacteria among children attending the outpatient department at the University of Gondar Comprehensive Specialized Hospital, Northwest Ethiopia

**Abstract**

- Emphasize antibiotic resistance findings: Highlight the significance of the methicillin resistance observed in *S. aureus* and *coagulase-negative staphylococci.*
- Strengthen the conclusion: In the conclusion, summarize the main findings succinctly and highlight their implications for public health.
- Highlight the practical implications of the findings and how they can inform clinical practices, policy decisions, and future research directions.

Study design, period, and area

- Include additional information about the hospital such as its specialty areas, facilities, and departments relevant to the study.
- Clarify the population size estimation: Explain the methodology used to estimate the total population of Gondar town as 2,929,628. Provide references or sources for this estimation to establish its validity.

Study population

- Explain why these departments were chosen for the study and how they are representative of the target population.

Specimen collection, transportation, and processing

- Explain the specific responsibilities of the trained laboratory technologist in data collection. Elaborate on their training and expertise to establish their credibility and reliability in accurately collecting the data.
- Mention any quality control measures taken during the data collection process.

**Results**

- Provide more context for previous hospital visits, respiratory tract infections, and antibiotic use: Expand on the significance of these factors in the study. Explain why previous hospital visits, respiratory tract infections, and antibiotic use within the last three months were relevant to the research question or hypothesis.
- Check exclusion criteria with antibiotic use within the last three months

**Discussion**

Discuss any potential biases, sample size considerations, or limitations in the methodology that may affect the generalizability or interpretation of the results.

Explore the implications: After discussing the findings, consider discussing the potential implications of the results. How do these findings contribute to the current understanding of nasopharyngeal carriage of Gram-positive bacteria? Are there any practical implications for healthcare practices, antibiotic stewardship, or future research directions?

**General comments**

Checked for clarity and consistency: Review the entire section for any grammatical errors, typos, or inconsistencies in the writing style. Ensure that the information is presented in a clear and organized manner
