# Supplementary material for: Nasopharyngeal carriage, antimicrobial susceptibility patterns, and associated factors of Gram-positive bacteria among children attending the outpatient department at the University of Gondar Comprehensive Specialized Hospital, Northwest Ethiopia
Source: PLoS One. 2024 Aug 28;19(8):e0308017. doi: 10.1371/journal.pone.0308017 (PMC11357108; doi:10.1371/journal.pone.0308017)
Supplement: Attachment — Submitted filename: Response to Reviewers (R1).docx [file pone.0308017.s005.docx]

**Rebuttal Letter**

**Date**: July 2, 2024

**Original Manuscript Number:** PONE-D-24-10619R1

**Original Article Title:** Nasopharyngeal Carriage, Antibiotic Susceptibility Patterns, and Associated Factors of Gram-positive Bacteria among children attending the outpatient department at the University of Gondar Comprehensive Specialized Hospital, Northwest Ethiopia

**To**: PLOS ONE

**Re**: Response to academic editor and reviewers

Dear Editor,

Thank you for allowing a resubmission of our manuscript, with an opportunity to address the academic editor and reviewers’ comments.

We are uploading

(1) Our point-by-point response to the comments below (response to academic editor and reviewer(s), respectively)

(2) A marked-up copy with track changes (Revised Manuscript with Track Changes)

(3) An unmarked version without track changes (Manuscript)

Best regards,

<Abebe Birhanu> et al

**NOTE: We have further addressed the Journal Requirements as follows:**

**Concern #1.** Please review your reference list to ensure that it is complete and correct. If you have cited papers that have been retracted, please include the rationale for doing so in the manuscript text, or remove these references and replace them with relevant current references. Any changes to the reference list should be mentioned in the rebuttal letter that accompanies your revised manuscript. If you need to cite a retracted article, indicate the article’s retracted status in the References list and also include a citation and full reference for the retraction notice.

**Authors’ Response:** We have thoroughly reviewed each reference and have not included any retracted articles.

**Authors' Response to Additional Editor Comments**

Dear Editor, we are grateful for the opportunity to revise our manuscript again for PLOS ONE and for the insightful comments from the reviewers, which have significantly improved our work.

**Editor Comment**: See the reviewer comments and the previous comments, still they require attention. Follow the binomial nomenclature of bacteria.

**Authors’ Response**: We thank you for highlighting this issue again. We have revised the manuscript to properly use the binomial nomenclature for bacterial names.

**Authors’ Response to Reviewer #1:**

First, we would like to extend our gratitude to the reviewers for their critical, constructive, and timely comments. To facilitate your review, we have copied your comments below alongside our responses. We have addressed all questions and concerns point by point as follows:

**Reviewer #1 Comment**: Thanks for addressing my concerns. As much as possible, the authors address and cover my concerns.

Authors’ Response: We thank you for providing good feedback.

**Authors’ response to Reviewer #2:**

**Reviewer #2 Comment**: The manuscript titled "Nasopharyngeal Carriage, Antibiotic Susceptibility Patterns, and Associated Factors of Gram-positive Bacteria among children attending the outpatient department at the University of Gondar Comprehensive Specialized Hospital, Northwest Ethiopia" presents a significant study addressing nasopharyngeal carriage and antibiotic resistance patterns in Gram-positive bacteria within a pediatric population. The study is well-organized with clear objectives, methodology, and findings that are valuable for understanding antimicrobial resistance in the region. The topic is highly pertinent given the rising concern of antibiotic resistance. The results offer important insights into the prevalence and resistance patterns of Gram-positive bacteria, crucial for guiding treatment strategies and public health measures.

**Authors’ Response**: We thank you for giving positive feedback.

**Reviewer #2 Comment**: While the introduction provides a solid background, it would benefit from a more comprehensive discussion on the global and regional implications of antibiotic resistance in Gram-positive bacteria. Including recent numerical data on antibiotic resistance trends would enhance the study's rationale.

**Authors’ Response**: We thank you for raising this issue. We have included the additional information in the introduction section as requested in the revised manuscript. Please see lines 108-113 of the manuscript.

**Reviewer #2 Comment**: Remove the objectives section stated at the end of the introduction: lines 104-115.

**Authors’ Response**: We have removed the objectives section from the end of the introduction as requested in the revised manuscript.

**Reviewer #2 Comment**: The “Methods” section is too lengthy and should be rewritten to conform to the journal guidelines. Please condense it accordingly.

**Authors’ Response**: We have revised and condensed the methods section to align with the journal guidelines.

**Reviewer #2 Comment**: The results section is well-written but should be minimized by focusing on the objectives. Table 1 size should be reduced to focus on socio-demographic characteristics, as this data is also presented in Table 5.

**Authors’ Response**: We have revised the results section to better align with the objectives and have minimized the size of Table 1 accordingly. Please refer to lines 261-268 of the manuscript.

**Reviewer #2 Comment**: The discussion effectively interprets the findings.

**Authors’ Response**: We thank you for providing good feedback.

**Reviewer #2 Comment**: The conclusion succinctly summarizes the main findings. However, please categorize your findings (e.g., prevalence data as low/medium/high) and provide more specific recommendations for future research and public health policies based on your findings.

**Authors’ Response**: We thank you for the suggestion. We have categorized the findings accordingly and added specific recommendations in the revised manuscript. Please see lines 470-482 of the manuscript.

**Reviewer #2 Comment**: The overall style of the manuscript is acceptable but does not fully comply with the PLOS One Journal standards. Please try to minimize the page of the manuscript by focusing on the main findings.

**Authors’ Response**: We have minimized the manuscript’s length and made the required corrections to adhere to the PLOS ONE Journal standards.

**Reviewer #3 Comment**: Confirm if consent was obtained from both minors and guardians, regarding publication ethics.

**Authors’ Response**: We confirmed that written informed assent and consent were secured from both the children and their guardians.

**Reviewer #3 Comment**: Confirm any potential competing interests.

**Authors’ Response**: We thank you for raising this issue again. The authors have declared that no competing interests exist and have approved the final submission of the revised manuscript.

**Title: - Nasopharyngeal Carriage, Antibiotic Susceptibility Patterns, and Associated Factors of Gram-positive Bacteria among children attending the outpatient department at the University of Gondar Comprehensive Specialized Hospital, Northwest Ethiopia**

**Abstract**

**Comment #1**: Emphasize antibiotic resistance findings: Highlight the significance of the methicillin resistance observed in *S. aureus* and coagulase-negative staphylococci. Strengthen the conclusion, summarize the main findings succinctly, and highlight their implications for public health. Highlight the practical implications of the findings and how they can inform clinical practices, policy decisions, and future research directions.

**Authors’ Response**: We thank you for pointing out the comment and we have made corrections accordingly. The significance of methicillin resistance observed in both *S. aureus* and coagulase-negative staphylococci has the following implications for public health, clinical practice, and future directions:

1. Methicillin-resistant *S. aureus* (MRSA) and methicillin-resistant coagulase-negative staphylococci (MRCoNS) are resistant to beta-lactam antibiotics, including methicillin and other penicillin derivatives. This resistance complicates treatment options for infections caused by these bacteria, as they are often resistant to multiple classes of antibiotics, limiting effective therapeutic choices.

2. MRSA and MRCoNS are associated with higher morbidity and mortality rates compared to methicillin-sensitive strains. They are often responsible for healthcare-associated infections (HAIs) and community-acquired infections, posing a significant burden on healthcare systems and increasing healthcare costs.

3. The presence of methicillin resistance underscores the importance of antibiotic stewardship programs to ensure appropriate use of antibiotics, minimize resistance development, and preserve the effectiveness of available antimicrobial agents.

4. MRSA and MRCoNS are notorious for their ability to spread easily in healthcare settings, leading to outbreaks. Effective infection control measures, including hand hygiene, isolation protocols, and environmental cleaning, are crucial to prevent transmission.

5. Continuous monitoring of methicillin resistance patterns in *S. aureus* and coagulase-negative *staphylococci* is essential for understanding epidemiological trends, guiding treatment strategies, and developing new therapeutic approaches, such as novel antibiotics or alternative treatment modalities.

**Study design, period, and area**

**Comment #2:** Include additional information about the hospital such as its specialty areas, facilities, and departments relevant to the study.

**Authors’ Response**: We have added additional information “The UoGCSH, a major teaching hospital with 977 beds, and 29 wards and emergency rooms, has served nearly 13 million people in its surrounding area and neighboring regions. It provides a wide range of medical services, including internal medicine, surgery, obstetrics and gynecology, pediatrics, laboratory tests, eye care, physiotherapy, dental care, cervical health, psychiatry, dermatology, and drug supply. The hospital also offers various social services and has specialized units for tuberculosis, kala-azar, cancer treatment, fistula surgery, psychiatric and psychological treatment, palliative and rehabilitation services, adult and pediatric intensive care unit, and cataract surgery” to the revised manuscript. We have attached source files as references in the “Other file type” section of the system. Please see it accordingly.

**Comment #3:** Clarify the population size estimation: Explain the methodology used to estimate the total population of Gondar town as 2,929,628. Provide references or sources for this estimation to establish its validity.

**Authors’ Response**: We appreciate your concern and have taken steps to address it by gathering updated information from local sources. As per your request, we have obtained information from the Central Statistical Agency of Ethiopia, Gondar branch, indicating that the estimated population of Gondar town in 2023 is 487,224. This estimation is obtained based on population projection methods utilizing data from the 2007 census data, as Ethiopia has not conducted a new census since that time. For detailed verification, we have attached supporting documents received from the Central Statistical Agency of Ethiopia, Gondar branch as references in the “Other files type” section of the system. Please refer to these files for further information.

**Study population**

**Comment #4:** Explain why these departments were chosen for the study and how they are representative of the target population.

**Authors’ Response**: We appreciate you for bringing this issue to our minds. Outpatient children were chosen for the study of nasopharyngeal carriage of Gram-positive bacteria for several reasons, making them representative of the target population effectively:

1. Outpatient children often visit clinics for respiratory infections, which makes them a suitable group for studying nasopharyngeal bacterial carriage. These infections are common in the general pediatric population, ensuring that the study captures a relevant and typical scenario.

2. Outpatient settings provide easier access to a diverse and large number of children compared to inpatient settings. This makes it practical to recruit participants and conduct the study without the constraints associated with hospitalized children, such as severe illness or hospitalization-related factors.

3. Outpatients generally represent less severe cases of illness compared to inpatients. This is crucial for studying nasopharyngeal carriage because it reflects bacterial colonization in a typical health condition rather than an extreme one, offering a more accurate picture of the bacteria present in the general child population.

4. Choosing outpatient children helps minimize selection bias. Hospitalized children might have been exposed to hospital-acquired infections or may have underlying conditions that could skew the results. Outpatients, on the other hand, are more likely to represent a healthy, broader pediatric population.

5. Outpatient clinics see children from a wide range of demographics, including different ages, socio-economic backgrounds, and health statuses. This diversity makes the sample more representative of the overall child population.

6. Outpatients typically live in the community and are thus more representative of the bacteria circulating in the community than those specific to hospital environments.

**Specimen collection, transportation, and processing**

**Comment #5:** Explain the specific responsibilities of the trained laboratory technologist in data collection. Elaborate on their training and expertise to establish their credibility and reliability in accurately collecting the data.

**Authors’ Response**: We thank you for pointing out this issue again. By "trained laboratory technologists," we refer to professionals who hold BSc degrees in medical laboratory sciences and MSc degrees in Medical Microbiology. In addition, they have extensive experience working in bacteriology laboratories at the University of Gondar Comprehensive Specialized Hospital. They are also certified by the Ethiopian Public Health Institute in standard bacteriology procedures and have experience as data collectors for many PhD and MSc research projects. In our study, these technologists were responsible for accurately labeling patient information, transporting samples to the laboratory under conditions that prevent degradation or contamination, and correctly interpreting and recording laboratory test results.

**Comment #6:** Mention any quality control measures taken during the data collection process.

**Authors’ Response**: We thank you for highlighting this concern. We understand that quality control is crucial for ensuring the accuracy, reliability, and validity of study results. In our study on the nasopharyngeal carriage of Gram-positive bacteria, we implemented the following quality control measures:

1. Trained nurses collected nasopharyngeal samples using sterile nasopharyngeal swabs and standardized procedures to ensure the outcome of the results.

2. A rigorous labeling system was used to accurately identify and trace each sample with the corresponding study participants throughout the study, with appropriate transport media and specified temperature maintenance to preserve sample integrity.

3. Standard bacterial reference strains were received from the Ethiopian Public Health Institute to verify the accuracy and reliability of the test results.

4. Double data entry was performed by different individuals, with any discrepancies resolved to ensure data accuracy.

5. Informed consent was obtained from all participants or their guardians, adhering to ethical guidelines.

**Results**

**Comment #7:** Provide more context for previous hospital visits, respiratory tract infections, and antibiotic use: Expand on the significance of these factors in the study. Explain why previous hospital visits, respiratory tract infections, and antibiotic use within the last three months were relevant to the research question or hypothesis.

**Authors’ Response**: We appreciate you for raising such an important comment. In a study on the nasopharyngeal carriage of Gram-positive bacteria in outpatient children, examining previous hospital visits, recent respiratory tract infections, and antibiotic use is highly significant for several reasons. These factors can influence the prevalence and types of bacteria present in the nasopharynx and help contextualize the study's findings. Here’s a more detailed explanation of why these factors are relevant to the research question or hypothesis:

Context and significance of previous hospital visits:

1. Hospitals are environments where patients are exposed to a variety of pathogens, including antibiotic-resistant strains. Children who have been hospitalized may carry different or more resistant bacteria compared to those who have not been exposed to such environments.

2. Hospitalized children often undergo various medical interventions, which can affect their microbiome. For instance, the use of intravenous lines, catheters, and other devices can introduce or select specific bacteria.

3. Hospital stays can impact a child's immune system either through the stress of illness or the treatments received. This can alter their susceptibility to colonization by different bacterial species.

Context and significance of respiratory tract infections:

1. Respiratory infections can disrupt the normal flora of the nasopharynx. Viral infections, in particular, can predispose to secondary bacterial infections, altering the composition and abundance of nasopharyngeal bacteria.

2. Inflammation caused by respiratory infections can change the local environment in the nasopharynx, potentially making it more hospitable to certain bacterial species that thrive in inflamed tissues.

3. During and after respiratory infections, children may have higher rates of bacterial transmission due to coughing and sneezing, which can affect the carriage rates observed in the study.

Context and significance of antibiotic use:

1. Antibiotic use is a major factor in selecting resistant bacterial strains. Recent antibiotic treatment can reduce the presence of susceptible bacteria and increase the prevalence of resistant strains, which is critical for understanding patterns of bacterial carriage and resistance.

2. Antibiotics can disrupt the normal microbial balance in the nasopharynx, leading to overgrowth of non-target organisms. This can result in an atypical bacterial community that might not reflect normal carriage patterns.

3. Following antibiotic treatment, the nasopharynx may be recolonized by different bacterial species, including opportunistic pathogens. This recolonization process can influence the types of bacteria found during the study period.

Relevance to research question or hypothesis

By understanding the impact of previous hospital visits, researchers can differentiate between community-acquired and hospital-acquired bacterial strains, providing insights into the sources and transmission dynamics of bacterial carriage in children. In addition, assessing the role of recent respiratory infections helps in understanding how such infections might predispose children to secondary bacterial colonization, influencing overall carriage rates and potentially identifying periods of increased vulnerability. Furthermore, analyzing the effect of recent antibiotic use is crucial for understanding how antibiotics shape the nasopharyngeal microbiome, the emergence of resistance, and the potential for recolonization by different bacteria.

**Comment #8:** Check exclusion criteria with antibiotic use within the last three months

**Authors’ Response**: We appreciate your careful observation of this issue. In our study, children who took any medication recently, mainly from 1 to 2 weeks before data collection, were excluded from the study to minimize the generation of false-negative results. However, If the timeline (gap) between the data collection period and the time of antibiotic exposure was beyond two weeks, the study participants were included in the study. We have excluded children who took antibiotics within this period for the following reasons:

1. Antibiotics can significantly disrupt the natural balance of the nasopharyngeal microbiome. This disruption can lead to a temporary reduction in bacterial diversity and abundance, skewing the results of the study.

2. Including children who have recently taken antibiotics might lead to an overestimation of resistance rates, which could distort the study’s findings on the prevalence of resistance in the general population.

3. After antibiotic treatment, the nasopharynx undergoes a period of recolonization. During this time, the bacterial population might not be stable, leading to variability that can confound study results.

4. Children who have used antibiotics recently are less likely to carry certain susceptible bacteria, potentially leading to biased carriage rates that do not accurately reflect the typical pediatric population.

**Discussion**

**Comment #9:** Discuss any potential biases, sample size considerations, or limitations in the methodology that may affect the generalizability or interpretation of the results.

**Authors’ Response**: We thank you for bringing this issue to our focus. We understand that addressing potential biases, sample size considerations, and methodological limitations that could impact the generalizability and interpretation of the results on the nasopharyngeal carriage of Gram-positive bacteria in outpatient children is crucial. Here are some detailed discussions on these aspects:

The participation of study participants in our study was voluntary base and families who choose to participate may differ systematically from those who do not. For example, more health-conscious parents might be more likely to consent to their child's participation, which may lead to skewing the results. In addition, while necessary to avoid recent antibiotic users, this exclusion criterion could introduce bias by excluding a subset of children who might have different carriage rates or bacterial profiles. Our study was also conducted in a specific geographic area and the findings may not be generalizable to populations in different regions with different environmental exposures and healthcare practices. Moreover, we used a cross-sectional design, which may not show trends in nasopharyngeal bacterial carriage rates and resistance patterns over time.

**Comment #10:** Explore the implications: After discussing the findings, consider discussing the potential implications of the results. How do these findings contribute to the current understanding of nasopharyngeal carriage of Gram-positive bacteria? Are there any practical implications for healthcare practices, antibiotic stewardship, or future research directions?

**Authors’ Response**: We thank you for reminding us of such an important point in our minds.

Our study findings have the following contribution to the current understanding of nasopharyngeal carriage of Gram-positive bacteria and implications for healthcare practices, antibiotic stewardship, or future research directions.

1. The study provides valuable baseline data on the prevalence and types of Gram-positive bacteria commonly found in the nasopharynx of outpatient children. This contributes to the epidemiological understanding of bacterial colonization in this demographic. It also gives insights into which species are most prevalent and can help in understanding the typical bacterial pathogen in children, influencing diagnostic and treatment approaches for respiratory infections.

2. By identifying the resistance patterns of nasopharyngeal bacteria isolates, the study highlights the extent of antibiotic resistance in hospital settings. This is crucial for guiding empirical treatment decisions and understanding the spread of resistant strains.

3. Findings can inform protocols for screening and monitoring nasopharyngeal carriage in children, particularly those with frequent hospital visits or recent antibiotic use. This can help in early identification and management of potential carriers of pathogenic bacteria. Moreover, an enhanced understanding of carriage dynamics can lead to better infection control measures in outpatient settings, reducing the spread of bacteria within the community.

4. The study supports the development of guidelines for rational antibiotic use in pediatric outpatient settings. By understanding common bacteria and their resistance profiles, healthcare providers can prescribe antibiotics more judiciously. In addition, findings can be used to inform educational programs for healthcare providers and parents about the importance of appropriate antibiotic use and the risks associated with overuse and misuse.

5. Future research could focus on longitudinal studies to understand how nasopharyngeal carriage of Gram-positive bacteria changes over time, particularly in response to antibiotic prescribing practices. Furthermore, incorporating molecular techniques like whole-genome sequencing can provide deeper insights into the genetic determinants of antibiotic resistance and the virulence factors of nasopharyngeal bacteria.

**General Comments:** Check for clarity and consistency: Review the entire section for any grammatical errors, typos, or inconsistencies in the writing style. Ensure that the information is presented in a clear and organized manner.

**Authors' Response:** Thank you for highlighting this issue. We have addressed grammatical errors, inconsistencies, typographical errors, and clarity issues in the revised manuscript to improve its consistency and readability.

Thank you very much for your valuable time to review and comment on our manuscript.

Authors
