# Supplementary material for: Nasopharyngeal carriage, antimicrobial susceptibility patterns, and associated factors of Gram-positive bacteria among children attending the outpatient department at the University of Gondar Comprehensive Specialized Hospital, Northwest Ethiopia
Source: PLoS One. 2024 Aug 28;19(8):e0308017. doi: 10.1371/journal.pone.0308017 (PMC11357108; doi:10.1371/journal.pone.0308017)
Supplement: Attachment — Submitted filename: Response to Reviewers (R2).docx [file pone.0308017.s006.docx]

**Rebuttal Letter**

**Date**: July 13, 2024

**Original Manuscript Number:** PONE-D-24-10619R2

**Original Article Title:** Nasopharyngeal Carriage, Antibiotic Susceptibility Patterns, and Associated Factors of Gram-positive Bacteria among children attending the outpatient department at the University of Gondar Comprehensive Specialized Hospital, Northwest Ethiopia

**To**: PLOS ONE

**Re**: Response to academic editor and reviewers

Dear Editor,

Thank you for giving us the chance to resubmit our manuscript and respond to the comments from the academic editor and reviewers.

We are uploading

(1) Our point-by-point response to academic editor and reviewer(s) comments below (Response to Reviewers).

(2) A marked-up copy with track changes (Revised Manuscript with Track Changes)

(3) An unmarked version without track changes (Manuscript)

Best regards,

<Abebe Birhanu> et al

**NOTE: We have further addressed the Journal Requirements as follows:**

**Concern #1.** Please review your reference list to ensure that it is complete and correct. If you have cited papers that have been retracted, please include the rationale for doing so in the manuscript text, or remove these references and replace them with relevant current references. Any changes to the reference list should be mentioned in the rebuttal letter that accompanies your revised manuscript. If you need to cite a retracted article, indicate the article’s retracted status in the References list and also include a citation and full reference for the retraction notice.

**Authors’ Response:** Thank you for this valuable comment. We have entirely reviewed each reference and properly cited them in the manuscript.

**Authors' Response to Additional Editor Comments**

Dear Editor, we are grateful for the opportunity to revise our manuscript again for PLOS ONE and for the insightful comments from the reviewers, which have significantly improved our work.

**Editor Comment**: The paper was significantly improved, still requires a critical revision and proofreading.

**Authors’ Response**: Thank you for your feedback. We have critically revised the paper accordingly.

**Editor Comment**: Lines 40-43: Correct the following as follows: *Staphylococcus aureus* was the most prevalent 122/424 (28.8%), followed by *Streptococcus pneumoniae* 92/424 (21.7%). Methicillin resistance was observed in 19/122 (15.6%) of *S. aureus* and 3/60 (5%) of coagulase-negative staphylococcus (CoNS) species. Inducible clindamycin resistance was 10/122 (8.2%) in *S. aureus* and 4/53 (7.5%) in coagulase-negative staphylococcus species.

**Authors’ Response**: Thank you for the correction. We have made the changes as requested. Please refer to lines 40-44.

**Editor Comment**: Italics the ‘P’ to refer p-value throughout the document.

**Authors’ Response**: Thank you for this important comment. We have italicized the letter "*P*" throughout the manuscript.

**Editor Comment**: The conclusion part of the abstract should contain concrete ideas and should emanate from the result based on your objective.

**Authors’ Response**: Thank you for bringing this to our attention. We have revised the abstract to include concrete ideas based on our objectives. Please refer to lines 50-61.

**Editor Comment**: Lines 63-65: It is better written as, …………. *S. pneumoniae*, *S. aureus*, coagulase-negative staphylococcus species (CoNS), and *Streptococcus pyogenes*.

**Authors’ Response**: We have accepted and corrected accordingly. Please check lines 66-67.

**Editor Comment**: Line 84 and 88: Write as, Coagulase-negative staphylococcus species………….

**Authors’ Response**: We have made the corrections as suggested. Please refer to lines 86 and 90.

**Editor Comment**: Line 119 and 120….…. this study addressed gaps left by prior research by offering data on the carriage of Gram-positive bacteria and their antimicrobial resistance patterns, including inducible……….

**Authors’ Response**: We have replaced "address" with "addressed" and "antibiotic" with "antimicrobial." Please see lines 122-123.

**Editor Comment**: It is preferable to use ‘antimicrobial’ than ‘drug’. For example, line 122 throughout the document. Uniformity is more important.

**Authors’ Response**: We have replaced the word "drug" with "antimicrobial" throughout the manuscript. Please refer to line 125.

**Editor Comment**: Unnecessary space between the heading and subheading should be removed.

**Authors’ Response**: Thank you for highlighting this. We have removed the unnecessary spaces between headings and subheadings.

**Editor Comment**: Line 125: The subheading ‘Study design, period, and area’. I recommend you to split into two subheadings as ‘Study area and setting’ and ‘Study design and period’

**Authors’ Response**: We have made the suggested changes. Please check lines 128 and 139.

**Editor Comment**: Line 135: Gondar town is located 750 km…….

**Authors’ Response**: We have added the verb "is" to the text. Please refer to line 137.

**Editor Comment**: Line 147: ‘Concisely’ was not the correct word.

**Authors’ Response**: We have changed "Concisely" to "Briefly" in the text. Please check line 151.

**Editor Comment**: Line 168: Specimen collection and processing

**Authors’ Response**: We have removed the word "transportation" as requested. Please see line 172.

**Editor Comment**: Line 181, 184: Write the source of the media (manufacturer/city/country) similar to line 191.

**Authors’ Response**: We have added the manufacturer and country "HiMedia, India" to the manuscript. Please check lines 185 and 188.

**Editor Comment**: Line 192: ……. Gram-positive diplococci….

**Authors’ Response**: We have corrected "Gram-positive cocci" to "Gram-positive diplococci" in the text. Please see line 191.

**Editor Comment**: How could you standardize the suspension preparation during performing antimicrobial susceptibility testing?

**Authors’ Response**: We appreciate this valuable comment. We have added the following to the revised manuscript: "To standardize the bacterial suspension preparation, 0.85% normal saline was used. If the suspension was more turbid than the 0.5 McFarland standard, it was diluted accordingly. Conversely, if the suspension was less turbid, additional pure colonies of the target bacteria were added to achieve the correct turbidity." Please refer to lines 215-218 in the revised manuscript.

**Editor Comment**: In the methods and result part, try to shorten the subheadings. For example, line 272, 279, and 299 can be written as follows, respectively: ‘Prevalence of Gram-positive bacteria’, ‘Antimicrobial susceptibility pattern’ & ‘Inducible clindamycin resistance’

**Authors’ Response**: We have made the requested changes. Please refer to lines 278, 287, and 306.

**Editor Comment**: Line 208: Antimicrobial susceptibility testing

**Authors’ Response**: We have corrected it as requested. Please refer to line 213.

**Editor Comment**: Line 227 and 228: ……… Results of antimicrobial susceptibility testing………………….

**Authors’ Response**: We have made the necessary corrections by replacing “antibiotic” with “antimicrobial”. Please see lines 233-234.

**Editor Comment**: Line 230: …………. multidrug resistance (MDR) [ 2]. Additionally, the reference you mentioned (reference 2) for MDR was not the correct reference/ and cannot be the source. It is a cross-reference and replace it by the correct reference.

**Authors’ Response**: Thank you for pointing this out. We have replaced "reference 2" with the correct reference (Magiorakos et al., 2012). Please refer to line 236.

**Editor Comment**: Line 257: A letter of permission was obtained from……………

**Authors’ Response**: We have replaced the word "acquired" with "obtained." Please check line 263.

**Editor Comment**: Line 309: Multi-drug resistance pattern

**Authors’ Response**: We have corrected it accordingly. Please see line 316.

**Editor Comment**: Line 324: Associated factors for nasopharyngeal carriage

**Authors’ Response**: We have corrected it as requested. Please check line 332.

**Editor Comment**: 421: The MDR proportion of 69.2%, 40%, 39.1%, and 18.2% were found in the current study for *S. aureus*…….

**Authors’ Response**: Thank you for the suggestion. We have revised it to: "The MDR proportions of 69.2%, 40%, 39.1%, and 18.2% were found in the current study for *S. aureus,* CoNS, *S. pneumoniae*, and *S. pyogenes*, respectively." Please refer to line 429.

**Editor Comment**: The conclusion and the recommendation were beyond your scope, so make it specific to your study.

**Authors’ Response**: We have revised the conclusion and recommendations to align with our findings and objectives. Please check lines 482-497.

**Editor Comment**: All tables and figure (s) should be cited in the text, otherwise it/they should be removed.

**Authors’ Response**: We have cited all tables and figures in the text at the end of their respective sentences. Please refer to lines 299, 337, and 347.

**Editor Comment**: Figure 1 should be prepared without description in tif and uploaded. The description should be placed within the text where you cited.

**Authors’ Response**: We have uploaded Figure 1 without description in "tif" format in the "figure section" of the system, and the description is placed within the text where it is cited. Please see lines 283-286.

**Authors’ Response to Reviewer #2:**

First, we would like to extend our gratitude to the reviewers for their critical, constructive, and timely comments. To facilitate your review, we have copied your comments below alongside our responses. We have addressed all questions and concerns point by point as follows:

**Reviewer #2 Comment:** The study is well-organized with clear objectives, methodology, and findings that are valuable for understanding antimicrobial resistance in the region. The topic is highly pertinent given the rising concern of antibiotic resistance. The results offer important insights into the prevalence and resistance patterns of Gram-positive bacteria, crucial for guiding treatment strategies and public health measures. The authors addressed all my concerns effectively. Thank you.

**Authors’ Response:** We thank you and appreciate your positive feedback.

Thank you very much for your valuable time to review and comment on our manuscript.

Authors
